# Effect of Caffeine Ingestion on Indirect Markers of Exercise-Induced Muscle Damage: A Systematic Review of Human Trials

**DOI:** 10.3390/nu14091769

**Published:** 2022-04-23

**Authors:** Leonardo Carvalho Caldas, Rafael Barreira Salgueiro, Neil David Clarke, Jason Tallis, Valerio Garrone Barauna, Lucas Guimaraes-Ferreira

**Affiliations:** 1 Postgraduate Program in Physical Education, Center of Physical Education and Sports, Federal University of Espirito Santo, Vitória 29075-910, ES, Brazil; leocaldas03@gmail.com (L.C.C.); barauna2@gmail.com (V.G.B.); ad6463@coventry.ac.uk (L.G.-F.); 2 Department of Physiology and Biophysics, Institute of Biomedical Sciences, University of Sao Paulo, Sao Paulo 05508-000, SP, Brazil; rafaeleefe@yahoo.com.br; 3 Centre for Sport, Exercise and Life Sciences, Coventry University, Coventry CV1 5FB, UK; neil.clarke@coventry.ac.uk; 4 Postgraduate Program in Physiological Sciences, Centre of Health Sciences, Federal University of Espirito Santo, Vitória 29075-910, ES, Brazil; 5 Postgraduate Program in Medical Sciences, Santa Cruz State University, Ilhéus 45662-900, BA, Brazil; 6 School of Life Sciences, Coventry University, Coventry CV1 5FB, UK

**Keywords:** ergogenic aids, recovery, lengthening contractions, muscle damage, delayed onset muscle soreness

## Abstract

The effect of caffeine on mitigating exercise-induced muscle damage (EIMD) is still poorly understood, but it was hypothesized that caffeine could contribute to decreasing delayed onset muscle soreness, attenuating temporary loss of strength, and reducing circulating levels of blood markers of muscle damage. However, evidence is not conclusive and beneficial effects of caffeine ingestion on EIMD are not always observed. Factors, such as the type of exercise that induces muscle damage, supplementation protocol, and type of marker analyzed contribute to the differences between the studies. To expand knowledge on the role of caffeine supplementation in EIMD, this systematic review aimed to investigate the effect of caffeine supplementation on different markers of muscle damage. Fourteen studies were included, evaluating the effect of caffeine on indirect muscle damage markers, including blood markers (nine studies), pain perception (six studies), and MVC maximal voluntary contraction force (four studies). It was observed in four studies that repeated administration of caffeine between 24 and 72 h after muscle damage can attenuate the perception of pain in magnitudes ranging from 3.9% to 26%. The use of a single dose of caffeine pre-exercise (five studies) or post-exercise (one study) did not alter the circulating blood levels of creatine kinase (CK). Caffeine supplementation appears to attenuate pain perception, but this does not appear to be related to an attenuation of EIMD, per se. Furthermore, the effect of caffeine supplementation after muscle damage on strength recovery remains inconclusive due to the low number of studies found (four studies) and controversial results for both dynamic and isometric strength tests.

## 1. Introduction

Caffeine consumption has been used as an ergogenic aid to improve exercise performance, as small but significant improvements were reported for long-duration aerobic exercise [1,2] and improving strength, muscle endurance, and power [3,4,5]. The low cost, easy acquisition, rapid absorption [6,7,8], and wealth of evidence supporting performance-enhancing effects, have contributed to the popularity of caffeine use in sports [9], with evidence estimating three out of four elite athletes (74%) use caffeine as an ergogenic aid before or during a sporting event [10].

In sports, even well-trained athletes could suffer from adverse effects caused by training sessions or competition, leading to muscle pain, temporary loss in muscle strength production capability, and reduced range of motion, which might impair recovery and sports performance [11,12,13]. In addition to improving sports performance, evidence also points out that caffeine could also contribute to attenuating exercise-induced muscle damage (EIMD) [14]. EIMD is characterized by physical damage to muscle fibers at the macro and micro structural levels, involving the sarcomeres, cell membrane, and connective tissue [15]. Eccentric muscle contractions are known to induce EIMD [16,17] but other factors also seem to influence EIMD occurrence, such as exercise intensity [18], skeletal muscle fiber type recruited during exercise [19], muscle contraction velocity [20], and joint range of motion during exercise [21,22]. EIMD is manifested by temporary impairments in muscle functioning, such as decreases in force production capacity, reductions in range of motion, swelling of the affected limb, increased stiffness, and muscle pain [23,24].

It was demonstrated that caffeine ingestion results in attenuation of delayed-onset muscle damage (DOMS) [25,26], attenuation of temporary loss of muscle [27] and reduction in blood markers of muscle damage [28]. However, evidence is not conclusive and beneficial effects of caffeine ingestion on EIMD are not always observed [29,30]. The differences between studies may be related to several possible factors, such as timing of supplementation (pre or post the event causing muscle damage), duration of supplementation protocol (e.g., acute versus chronic), and methods used for assessing muscle damage (e.g., pain perception, loss of strength, and blood markers of injury). Several studies have investigated the effect of caffeine using only one indirect marker of muscle damage, although it is not clear whether caffeine has a direct effect on each of these markers or if there is an interaction between them. For example, would decreases in pain perception with caffeine supplementation also be related to lower strength losses and lower circulating levels of creatine kinase after EIMD? Or do these effects happen independently?

Therefore, the objective of this literature systematic review is to expand the understanding of the role of caffeine in mitigating the damage related to muscle damage, investigating the mechanisms of action involved and how the different supplementation protocols can interfere with its effects on EIMD attenuation or recovery. In our understanding, there are two possibilities for caffeine’s actions on EIMD: (1) caffeine used before a muscle damage-inducing protocol could result in less muscle damage; and/or (2) caffeine consumption after induction of muscle damage could act to relieve symptoms, especially recovery in contractile function after EIMD.

## 2. Materials and Methods

### 2.1. Experimental Approach to the Problem

A systematic review was conducted following the procedures outlined by the Cochrane Handbook [31]. To guide the search, the following question was elaborated: Can caffeine supplementation attenuate exercise-induced muscle damage? The electronic searches included four recognized databases: PubMed, Scopus, Cochrane, and Bireme.

### 2.2. Procedure

The systematic literature search was performed using the following terms and Boolean operators: “caffeine” AND “muscle damage” OR “exercise induced muscle damage” OR “soreness” OR “delayed onset muscle soreness” OR “pain”. No restrictions on any language or year of publication were applied, but only articles with abstracts in English were included. The search was performed in January 2022. Inclusion criteria were studies with human participants that investigated the effects of any form of caffeine ingestion on direct muscle damage markers (e.g., using muscle biopsy or magnetic resonance imaging) and/or indirect markers (changes in maximum isometric strength, muscle pain, changes in joint range of motion, changes in muscle circumference indicating swelling and blood markers of muscle damage). Studies were excluded if they did not include a placebo condition for comparison or combined caffeine with other nutritional supplements. Additionally, studies using animal models were also excluded.

**Figure 1:**
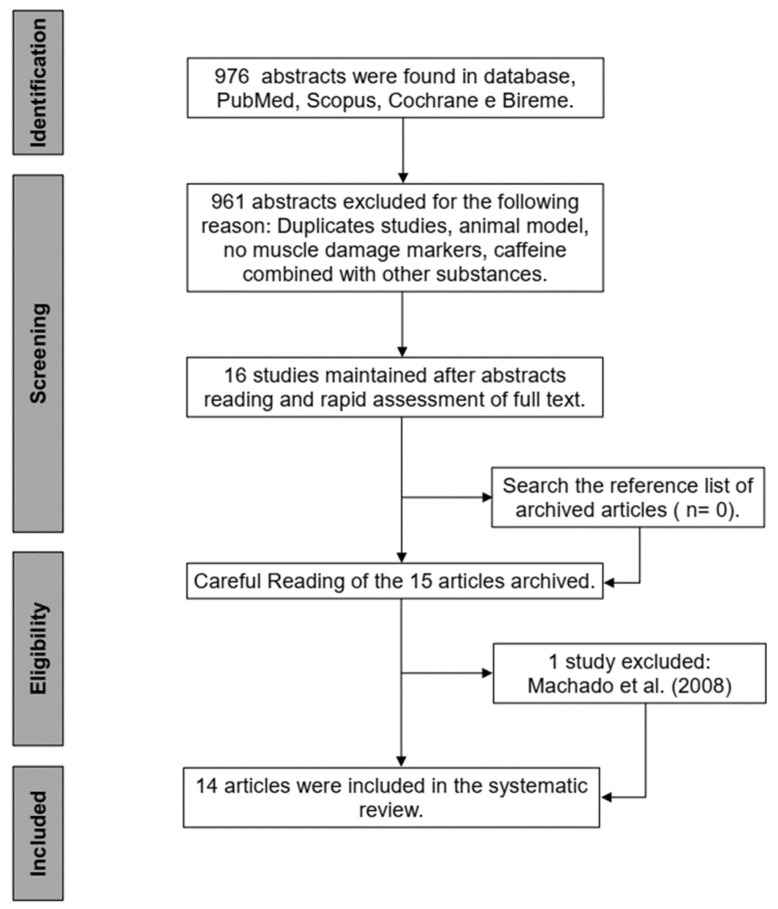
Procedure for selection of the studies and decision-marking inclusion and exclusion.

Following initial screening of the abstracts, two researchers independently screened the study’s full texts against the inclusion criteria. Any disagreements were discussed and resolved by consensus between both researchers. The literature search was performed in the following sequence: (A) the studies were electronically saved for later reading and evaluation; (B) initial screening was performed, abstracts were read, and those that did not meet the inclusion criteria were excluded; (C) the abstracts containing sufficient information described in the inclusion criteria and presented no reason for exclusion were archived for later reading of the full text; (D) after reading the full text, the studies were included or excluded according to the previous selection criteria; (E) after reading the full text from each study, the reference lists were also searched for any additional articles that were not found by our search strategy (Figure 1).

### 2.3. Coding and Classifying Variables

The main coded categories of each included study were: (a) identification of the studies (authors and year of publication); (b) characteristics of the sample (age, sex, level of training); (c) caffeine consumption habits of the participants; (d) characteristics of the methodological quality of the studies (randomization and blinding strategy, study design, allocation concealment, intervention monitoring, loss to follow-up); (e) muscle damage protocol; (f) caffeine supplementation protocol (dose administered and duration of supplementation). The characteristics of the studies are shown in Table 1.

Study quality was assessed according to the PEDro scale (https://pedro.org.au/english/resources/pedro-scale/ (20 April 2022)). The PEDro scale has strong reliability and validity [41,42,43] and has been widely used in other systematic reviews related to caffeine supplementation [2,5,44,45,46,47]. The scale consists of a list of 11 items; for each individual criterion of scientific methodology, studies receive a score of 1 when the criterion is clearly met or 0 when that criterion is not adequately met. The first item (eligibility criteria) does not receive a score because it is related to external validity and, therefore, does not reflect the quality dimensions assessed by the PEDro scale. Thus, the total scores range from 0 to 10. The criteria included in the Scale are criteria of: (1) eligibility criteria were specified (no score); (2) subjects were randomly allocated to groups; (3) allocation was concealed; (4) the groups were similar at baseline regarding the most important prognostic indicators; (5) there was blinding of all subjects; (6) there was blinding of all therapists who administered the therapy; (7) there was blinding of all assessors who measured at least one key outcome; (8) measures of at least one key outcome were obtained from more than 85% of the subjects initially allocated to groups; (9) all subjects for whom outcome measures were available received the treatment or control condition as allocated or, where this was not the case, data for at least one key outcome were analyzed by “intention to treat”; (10) the results of between-group statistical comparisons are reported for at least one key outcome; and (11) the study provides both point measures and measures of variability for at least one key outcome. The PEDro score for each study is shown in Table 2.

## 3. Results

### 3.1. Studies Search and Selection Process

The initial search (PubMed, Scopus, Cochrane, and Bireme) resulted in 976 articles; 961 of them were excluded after reading the title and abstract for not meeting the inclusion criteria. The remaining 15 articles were included for full-text screening; 1 study was excluded [48], as it was identified that another study [36] already included in this review, presented analogous participants and results. Therefore, at the end of the search, 14 studies [25,27,28,29,30,32,33,34,35,36,37,38,39,40] were included in this systematic review (Figure 1).

### 3.2. Studies and Participant Characteristics

All data characteristics of the studies could be found in Table 1. The sample size of each study ranged from 6 to 35 participants resulting in a total sample of 248 participants, 174 men (70%) and 74 women (30%); 127 participants (51%) were athletes from various sports (soccer, handball, basketball, cycling, skiers), 51 participants (21%) were physically active, 12 participants (5%) had experience with resistance training, 10 participants (4%) had no exercise experience, and 48 participants (19%) had no described training status.

There is no consensus on the classification of habitual caffeine consumption, but Filip et al. [49] proposed a classification based on daily caffeine intake relative to body weight (mg·kg^−1^.day^−1^). According to these authors, mild consumers ingest less than 3 mg·kg^−1·^day^−1^ of caffeine. Most of the studies included in the present review used absolute doses when accessing caffeine daily consumption, so we established the daily dose of 200 mg·day^−1^ as the limit between low/mild and moderate/high consumers, which is close to the values proposed by Filip et al. [49] if we consider a 70 kg adult. Eight studies [27,28,30,34,36,37,38,39] included participants with a history of low/mild caffeine consumption (<200 mg/day) representing 117 participants (47%); however, in the study by Hurley et al. [34], the participants’ mean caffeine consumption was not described. Three studies [25,29,33] included moderate to high caffeine users (>200 mg/day) representing 59 participants (24%) and three studies [32,35,40] did not assess mean caffeine consumption of participants, representing 72 participants (29%). Eleven studies (78.5%) used a double-blind cross-over design. In nine of those studies, the washout period between the caffeine/placebo condition was greater than 6 days and in two studies this period was less than 48 h. The other three studies (21.5%) opted for a double-blind design with parallel groups.

The protocol for inducing muscle damage was varied, ranging from exercises in isolated muscle groups (e.g., quadriceps and elbow flexors) to multi-articular exercises involving the whole body. Resistance exercise with eccentric muscle contraction for inducing muscle damage was used by six studies [29,30,33,34,38,48], and eight other studies [25,27,28,32,35,37,39,40] used a variety of test protocols including exercise on cyclergometer, treadmill running, stair climbing, and sport-specific movements for soccer and skiing.

Caffeine dosage varied from 3 mg·kg^−1^ to 7 mg·kg^−1^. Eight studies [28,32,35,37,38,39,40,48] used a single dose 55 to 70 min prior to an EIMD protocol. One study [27] used a single dose 24 h after the EIMD protocol and in five studies [25,29,30,33,34] caffeine was ingested two or more times between 48 and 72 h after EIMD.

### 3.3. Methodological Quality of Studies

The PEDro scale was used to assess the methodological quality of the studies (Table 2) and 12 studies (86%) received the maximum score (10 points). Work by Bassini-Cameron et al. [32] study received 8 of 10 total points, not fulfilling the seventh criterion, since three of the participants had been reassigned to the control group after protocol intervention for not completing the task; and it also affects the eighth criterion, in which the reallocation represented a loss of more than 85% allowed by this criterion. Maridakis et al. [30] scored nine points for not meeting the third criterion of the PEDro scale. The reason is that, even before caffeine treatment, the supposed caffeine treatment group already had a difference in pain perception vs. placebo group.

### 3.4. Effect of Caffeine Supplementation on Indirect Markers of Muscle Damage

Six studies (43%) evaluated the effect of caffeine supplementation on delayed onset muscle soreness (DOMS). Of these, four studies found that caffeine ingestion was able to reduce pain perception between 24 and 48 h following the muscle damage protocol [25,27,30,34], one study [33] found no significant difference between groups and one study [39] observed higher DOMS in the caffeine group when compared to the placebo group. In total, nine studies (64%) evaluated blood markers of muscle damage, two studies found higher circulating levels of creatine kinase (CK) in the caffeine-supplemented groups [32,39], and one study found the opposite result [28] with a higher circulating level of CK in the placebo group. Most studies (six studies) found no significant difference between groups (caffeine vs. placebo) for blood markers of muscle damage including CK, lactate dehydrogenase (LDH), aspartate aminotransferase, alanine aminotransferase, and oxidative stress markers, such as: malondialdehyde and total antioxidant capacity [34,35,36,37,38,40].

Four studies evaluated the effects of caffeine supplementation on maximum voluntary isometric contraction (MVIC) loss after EIMD. Chen et al. [27] observed that caffeine ingestion resulted in an attenuation of MVIC loss 48 h after the EIMD protocol. However, three other studies did not observe any difference between caffeine and placebo conditions on MVIC recovery after EIMD [29,30,33].

## 4. Discussion

The current systematic review aimed to summarize the effects of acute caffeine ingestion on attenuation of muscle damage or improving recovery after EIMD. Studies supplemented caffeine pre and/or post exercise-induced muscle damage protocol and evaluated at least one muscle damage marker. Due to the complexity of measuring muscle damage, studies have used a wide variety of direct and indirect markers [50,51]. Indirect markers used in the included studies were force production capacity loss, reduced joint range of motion, muscle swelling (increased limb circumference), DOMS, and blood markers (i.e., muscle damage, inflammation, and oxidative stress markers) [23,50,52]. Direct methods for assessing muscle damage included muscle biopsy and magnetic resonance imaging [51]. However, no studies included in the current review evaluated direct markers of muscle damage. All fourteen studies included in the systematic review evaluated the effects of caffeine on indirect markers of muscle damage. Nine studies included blood markers, six studies used pain perception, and four studies evaluated muscle strength loss after an EIMD protocol.

### 4.1. Muscle Soreness

The most common symptom of muscle damage induced by eccentric contractions is DOMS and it is also the most widely used injury marker in human studies [50,53,54]. It is postulated that caffeine has an analgesic effect on DOMS due to its action on the central nervous system [30,34,55]. Of six studies evaluating the effects of caffeine supplementation on DOMS, four observed a lower pain perception in caffeine condition when compared to placebo between 24 and 72 h after exercise. The magnitude of pain attenuation varied according to the scale used. Caldwell et al. [25], for example, observed reductions of 1.3 points in the caffeine group 24 h after exercise using a 1–6 point pain perception scale, which represents a reduction of 26%. In another study [34], pain in the caffeine group was reduced by 0.9 points 48 h after exercise, using a 0–10 point scale (9%), and Chen et al. [27] used a scale of 0–100 points and observed reductions of 11.2 points (11.2%) in the perception of pain in the caffeine group 48 h after exercise. Maridakis et al. [30], using the same scale, observed reductions of 3.9 points in the caffeine group 24 h after muscle damage induction exercise. It is important to highlight that in the Maridakis et al. study, pain perception analysis was performed while the participants were performing submaximal and maximum isometric eccentric muscle actions, in contrast to other studies where pain perception was accessed at rest or after exercise using muscle palpation.

Only two studies showed an absence or negative effect of caffeine on pain perception. Stadheim et al. [39] investigated the effects of a single dose of caffeine followed by a 10-min cross-country skiing exercise protocol on an ergometer. These authors observed that the caffeine groups showed elevated pain perception after 24 h of the test simultaneously with greater workload performed (longer distance covered) on the ergometer when compared to the placebo group. Therefore, the higher pain perception may be related to the injury caused by the greater effort during the ergometer test. Green et al. [33] evaluated pain perception 24 h after eccentric contractions of the knee extensor muscles and the effects of caffeine ingestion. The authors observed no significant differences in pain perception after the exercise protocol between caffeine and placebo groups. In this study, participants were regular caffeine consumers (although the exact daily caffeine intake was not reported), which differ from the four other studies that observed a decrease in pain perception after caffeine ingestion compared to placebo, using low caffeine consumers (daily caffeine consumption lower than 230 mg·kg^−1^ of body weight). Caffeine effect on habitual and non-habitual consumers remains under debate due to a limited number of studies and controversial results [9].

Taken together, four out of five studies using caffeine supplementation after the induction of EIMD showed decreases in pain perception after 24 to 72 h with magnitudes ranging from 3.9% to 26% when compared to the placebo group. It is also important to note that the peak in DOMS also occurred 48 to 72 h after EIMD [23,54,56]. Differences observed in the magnitude of DOMS attenuation after caffeine supplementation may be related to different exercise protocols, which could lead to different levels of muscle damage and due to the complexity of cellular events associated with muscle damage and the interaction of several chemical mediators after eccentric exercise capable of stimulating nociceptors.

It is proposed that DOMS is mainly caused by eccentric muscle contractions, which might lead to tissue microdamage and stimulate the release of chemokines from damaged myofibrils [15]. Chemokines enter the circulation and recruit inflammatory cells, which infiltrate skeletal muscle and release chemical mediators, such as bradykinins and prostaglandins. Bradykinins and prostaglandins act as signaling mechanisms by stimulating muscle fibers to synthesize neural growth factor (NGF) and glial derived neutrophil factor (GDNF), which are capable of stimulating nociceptors III and IV [54,57,58]. The bradykinins release can even occur in the absence of muscle cell damage and inflammation [58]. Alternatively, eccentric muscle contractions can cause high compressive forces within the muscle spindle, generating microdamage in type II afferent neurons that are also related to DOMS [59,60].

Another mechanism related to pain stimuli involves adenosine receptors. Those receptors are mainly regulated by ATP metabolism [61]. Adenosine levels increase in muscle and plasma during muscle contraction [62]. After eccentric exercise, the adenosine receptor 1 (A1) gene expression also increases, reaching approximately six-fold in skeletal muscle [63]. A1 receptors are involved nociception and pain processing located in several neural tissues, including the peripheral afferent nerves at the spinal dorsal horn level, as well as central areas, such as the brain cortex, cerebellum, and hippocampus [64]. The analgesic effect of caffeine may be related to its action as a non-selective adenosine antagonist blocking the pain perception, which is propagated from peripheral nerves to the central nervous system [30,34,55].

### 4.2. Muscle Strength Assessment

Measurements of maximum voluntary contraction are considered the best method to identify muscle damage, as it is directly related to the magnitude and change in the temporal course that occurs after the injury [50]. Despite this, our review identified only four studies (28%) that investigated the effects of caffeine on changes in MVIC after muscle injury. Chen et al. [27] performed a downhill running protocol on a motorized treadmill at 70% of VO2 max for 30 min with 10 male and 10 female athletes. Participants received a 6 mg·kg^−1^ bodyweight of caffeine or placebo 24 or 48 h after the EIMD protocol. Knee extensor MVIC, pain perception and blood CK levels were used as muscle injury markers. As hypothesized, there were reductions in knee extensor MVIC 24 and 48 h after the exercise protocol. Furthermore, caffeine-supplemented group also presented 10.2% of attenuation in MVIC loss 48 h after EIMD when compared to placebo ingestion.

Green et al. [33] evaluated the effect of caffeine supplementation (6 mg·kg^−1^) under two conditions: (a) muscle uninjured; (b) muscle injured. The sample consisted of eight men and eight women. Muscle damage was assessed using the pain perception scale (1–100 points) and two maximum strength tests using an isokinetic dynamometer: MVIC and maximum voluntary dynamic contraction (MVDC). In the muscle injured condition, muscle damage was induced by 100 eccentric quadriceps contractions and reassessed 24 h after exercise. As expected, in the muscle injured condition, reductions in strength performance were observed in both tests. The caffeine group had a smaller reduction in MVDC (−9.4%) when compared to the placebo group, but no difference was observed in the MVIC test. Similarly, in the muscle uninjured condition, caffeine ingestion did not improve MVIC test, but MVDC was improved by 6.8%. These results suggest that caffeine supplementation after an EIMD protocol results in the attenuation of dynamic strength loss after EIMD and increases dynamic strength performance in no injured muscle. However, isometric strength was not affected by caffeine supplementation in neither condition.

Another study [29] investigated the effect of caffeine supplementation (6 mg·kg^−1^) under two conditions, muscle uninjured and muscle injured. Participants performed 50 eccentric quadriceps contractions to induce muscle damage and MVIC was assessed 24 and 48 h after this protocol. MVIC performance was decreased during this period, but no differences were observed between caffeine and placebo conditions. When no EIMD was induced, caffeine resulted in a 10.4% improvement in MIVC performance and a 6.2% increase in muscle activation compared to placebo. In contrast to Green et al. [33], this study suggested that the positive effect of caffeine supplementation on MVIC only occurs in muscle without EIMD.

Maridakis et al. [30] investigated the effect of pre- and post-exercise caffeine supplementation (5 mg·kg^−1^), with a muscle damage induction protocol of 64 eccentric quadriceps contractions, performed by 10 women with no experience in resistance training. Muscle damage was assessed by pain perception (0–100 point scale) and quadriceps MVIC test performed 24 and 48 h after exercise. A decrease in MVIC after EIMD was observed but with no differences in strength and pain perception between caffeine and placebo conditions.

Taken together, the studies on the effect of caffeine on strength recovery after muscle injury have conflicting results. Two studies observed positive effects of caffeine supplementation after muscle damage but used different strength assessment methods (MVIC [27] or MVDC [33]). When analyzing the effect of caffeine ingestion after the EIMD protocol (supplementation post-EIMD) using isometric strength testing, three studies indicate that caffeine does not attenuate strength loss caused by muscle damage [29,30,33]. In the condition of uninjured muscle (supplementation without EIMD), a larger set of studies has observed that supplementation with caffeine enhances strength performance in different types of dynamic tests (e.g., maximal or submaximal isokinetic, 1 RM, vertical jump) [65,66].

The mechanisms involved in the decrease of strength performance after muscle damage seem to be related to impaired excitation–contraction coupling caused by disruptions that occur in the sarcoplasmic reticulum, transverse tubules, and sarcolemma [67]. On the other hand, caffeine could have positive effects on strength and power activities due to increased recruitment of motor units and improved excitation–contraction coupling [33,62]. Studies using high doses of caffeine in skeletal muscle cells isolated from an animal model observed direct effects, such as (1) increased calcium mobilization from the sarcoplasmic reticulum; (2) greater direct sensitivity to calcium in skeletal muscle; (3) modifications in Na+/K+ ATPase activity [68,69]. Caffeine concentrations used in cell culture studies are considered toxic when extrapolated to human studies [27,62,70]. However, it was demonstrated that micromolar concentrations of caffeine can result in a small but significant enhancement in power output (3–6%) in isolated mouse skeletal muscles [68]. In humans, blood caffeine concentrations reaches 10 to 70 uM after the ingestion of 3–9 mg·kg^−1^ [71]. Based on the available data, the possibility of a direct effect of caffeine on skeletal muscle (damaged or intact) with doses commonly used in studies employing human participants cannot be discarded. Therefore, a possible mechanism for the effects of caffeine on the recovery of muscle function following EIMD is the attenuation of the impaired excitation–contraction coupling after the muscle damage by its direct action on skeletal muscle increasing calcium sensitivity, and also attenuating extracellular K^+^ accumulation due to increased Na^+^/K^+^ ATPase activity in skeletal muscle.

Another suggested mechanism is the effect of caffeine on the CNS reducing pain perception (see topic Section 4.1). It is well accepted that nociceptive stimuli reduces motor cortical excitability [27,72]. Chen et al. [27], for example, observed an inverse correlation between muscle pain intensity and strength production capacity. In addition, caffeine supplementation contributed to pain relief and strength recovery after muscle injury. However, this evidence was not confirmed by other studies, such as Maridakis et al. [30]. After muscle damage, authors observed a reduction in pain perception in individuals treated with caffeine; however, it was not enough to attenuate MVIC strength losses. Green et al. [33] observed greater recovery of MVDC in the caffeine group, although the pain perception was not different between the caffeine and placebo groups. Therefore, it remains only speculative that pain may interfere with the ability to produce force and some evidence suggests that damaged muscles could be fully active regardless of the muscle pain existence [51,73].

### 4.3. Blood Markers of Muscle Damage

The presence of muscle proteins and enzyme fragments in the bloodstream is indicative of muscle damage [51]. Muscle injury is characterized by damage to the extracellular matrix resulting in loss of sarcoplasmic membrane integrity, allowing muscle proteins to leak from the cell into the circulation, such as CK, hemoglobin, LDH, and several others [24,51,74]. Although several muscle proteins can be used as indirect injury markers, CK has received more attention, once the magnitude of its increment is larger relative to other proteins and the cost of the assay is comparatively lower [51]. For example, in this review, nine studies that used blood markers, used at least CK as one of the markers of muscle damage.

However, despite being widely used in studies, the use of blood CK as an indirect marker of muscle damage is controversial and is not necessarily related to the magnitude of structural damage in skeletal muscle cells. For example, Fielding et al. [75] observed no relationship between blood CK levels and Z-Band ultrastructural damage after eccentric contractions of the quadriceps muscle. Additionally, the authors observed a greater increase in CK in the group that received fluid and electrolytes replacement during exercise, demonstrating that the hydration level can influence the CK response to the same exercise protocol. In addition, other factors such as gender and age can also influence plasma CK concentrations, as discussed by Baird et al. [76]. Therefore, the results of studies that used blood markers of muscle damage, such as CK, should be interpreted with caution.

The effect of caffeine on markers of muscular damage in the bloodstream is still poorly understood, and some studies suggest that it could increase the inflammatory response due to its antagonistic action on adenosine receptors. By binding to A2A and A2B receptors on immune system cells, adenosine inhibits their activity, reducing the inflammatory cells infiltration and the pro-inflammatory cytokines expression [77]. Caffeine ingestion could abrogate adenosine receptor signaling and increase the acute inflammatory response contributing to increased muscle damage [32,77].

Nine studies investigated the effect of caffeine supplementation on muscle damage, with eight studies using a single dose before the EIMD protocol, and only one study providing caffeine on several moments after muscle damage induction [34]. In six studies, no differences in circulating levels of CK or LDH were observed between caffeine and placebo conditions (five with pre-EIMD supplementation and one post-EIMD). Only three studies observed differences between conditions, all providing a single caffeine dose prior to EIMD. Bassini-Cameron et al. [32] and Stadheim et al. [39] observed a higher CK level in the caffeine group, while Ferreira et al. [28] observed the opposite. To induce muscle damage, these last three studies used maximum effort exercise protocols (all-out type), which do not allow equalizing the work volume between groups. Therefore, the circulating CK level may be related to the greater work performance during the test and not to the direct effect of supplementation in inducing muscle damage. For example, in the study of Stadheim et al. [39], the highest CK levels in the caffeine groups were accompanied by the greater volume of work performed by the caffeine group. The other two studies, Bassini-Cameron et al. [32] and Ferreira et al. [28], did not report whether there was a difference between groups for workload in the Yo-Yo test or in the repeated sprints test on the cycle ergometer, respectively. Taken together, the research does not appear to demonstrate that caffeine supplementation results in increased muscle damage after an exercise bout, but due to a limited number of studies, future research is needed to better clarify this issue.

However, caffeine may modulate the anti-inflammatory response. It was demonstrated that acute supplementation of 6 mg·kg^−1^ of caffeine before completing a 15 km race improves the anti-inflammatory response indicated by increases in plasma levels of IL-6 and IL-10 [78]. This finding is particularly interesting and could explain a possible protective effect on injured skeletal muscle. The EIMD is divided into two phases, the first characterized by the result of eccentric muscle actions causing damage to the muscle fiber and cellular matrix. The second phase is characterized by activation of proteolytic pathways mediated by Ca^2+^, migration of inflammatory cells to the injured site, and production of reactive oxygen species that contribute to further increase muscle damage [24,56]. In a study with an animal model, it was observed that attenuating the inflammatory process related to the second phase of muscle damage with the use of non-steroidal anti-inflammatory drugs improves recovery from EIMD, with an attenuation of strength deficit following an eccentric contraction protocol [79]. Therefore, future studies should investigate whether caffeine could have similar anti-inflammatory effects and could contribute to reducing secondary muscle damage.

### 4.4. Methodological Considerations

Based on the PEDro scale, 12 studies (86%) received the maximum score (10 points) and only 2 studies (14%) received a score between 8 and 9 points; therefore, all included studies, according to the scale, are classified as good or excellent [5,44].

Other important methodological issues affecting study results are highlighted in Table 2, including participants’ average caffeine consumption history, type of study design, muscle damage induction protocol, and supplementation protocol. Regarding the methodological design, 3 studies opted for a design with parallel groups (independent samples) while the majority (11 studies) used a cross-over design (dependent samples). The parallel-type design has some disadvantages, for example, it has been observed that subjects undergoing the same muscle damage protocol respond differently. Lower responders reduce MVC test by 18.5%, while higher responders lose muscle MVC strength by 57.8% [23]. Individual variability has also been observed for the effect of caffeine supplementation on performance [80]. Cross-over designs may be more useful to reduce individual variability observed both related to muscle damage and caffeine.

For specific studies that used the cross-over design, the wash-out period between the two conditions tested could also affect the results. Two of them used relatively short intervals between conditions (caffeine or placebo) (between 24 and 48 h). Although the caffeine effect had a short half-life (4 to 6 h), the duration of muscle damage effects could last for several days [15,23,81]. The investigation of these two factors in a short period of time could generate misleading results. For example, if the investigation of pain perception in the caffeine condition was carried out 48 h after exercise and the followed placebo experimental condition, without correct washout, it is possible that an increased pain perception will not be directly related to the supplementation condition, but rather to the pain perception evolution peak, which usually occurs between 48 and 72 h after muscle damage. To avoid this, it would be necessary to ensure complete recovery of muscle injury markers before subjecting them to a second condition.

Regarding the supplementation protocol, the dose used ranged from 3 to 7.5 mg/kg of body weight, which corresponds to the dosage widely used in the literature with positive effects on sports performance [9]. The majority, nine studies (64%) investigated the effect of supplementation for a short period of time (<24 h after muscle injury), while only five studies (36%) investigated the effect for a longer period than 48 h after muscle injury. Considering that, the effects of muscle injury could last for longer periods (7 days), longer-term studies in the monitoring of muscle injury markers should be recommended to investigate the effect of caffeine on muscle recovery.

To avoid bias factors, the muscle damage induction protocol must also ensure the volume and intensity equalization for both groups evaluated. Some studies used exercise protocols that did not guarantee this equalization. Caldwell et al. [25] used a time trial as test; Bassini-Cameron et al. [32] tested the volunteers until fatigue, and some studies performed a maximal effort test [28,37,38,39]. In all of these uncontrolled studies, there exist the possibility of one of the groups exerting greater effort during the test than the other group, leading to a further higher muscle injury level, masking the effect of supplementation on muscle recovery. In addition, five of those studies performed supplementation with caffeine in the pre-exercise moment, which could contribute to higher performance of the caffeine groups during the test and higher levels of muscle damage.

### 4.5. Limitations and Future Perspectives

In this systematic review, 14 studies were found that evaluated the effect of caffeine on indirect muscle damage markers. It is worth mentioning that indirect muscle damage markers present limitations and significant differences on magnitude and time course responses among them [50,51]. Other limitations when using indirect markers include: (1) although DOMS is often used as a marker of muscle damage, it can occur independently of the EIMD, and may even manifest in the absence of muscle damage [58]. (2) The use of blood activity of muscle enzymes to assess muscle damage is controversial. For example, it has been observed that serum CK concentrations after muscle damage are not correlated with other injury markers, such as muscle strength and DOMS [82] and, therefore, CK could be more useful as a qualitative marker of skeletal muscle trauma rather than a quantitative indicator of the extent of muscle damage [83]. (3) Maximum voluntary contraction peak torque is considered the best method to assess EIMD [50], but was used in only four studies. In addition, these studies presented controversial results possibly due to different strength assessment methods used. Furthermore, it is unclear whether the effect of caffeine on muscle strength recovery observed in some studies [27,33] was due to a protective effect against muscle damage, involving as yet unknown mechanisms of action. Most likely, caffeine ingestion results in strength loss attenuation following EIMD due to its ergogenic effects as discussed herein. Therefore, future studies using direct and indirect markers and different types of strength tests (isometric/dynamic) may contribute to understanding the role of caffeine in the prevention of damage and in the recovery of symptoms related to muscle damage. Another important consideration is related to the timing of caffeine ingestion. It is possible to consider two hypotheses for caffeine supplementation (which may even occur simultaneously). The first is that caffeine ingestion before an EIMD protocol could attenuate the occurrence of muscle damage. Another hypothesis is that the use of caffeine after a muscle damaging protocol could contribute to a better recovery from muscle damage, improving contractile function. Further studies should specifically address these issues to better understand the application of caffeine supplementation in EIMD.

Few studies investigated the effect of prolonged caffeine supplementation during the muscle recovery phase. Ideally, a well-designed study would constantly follow-up the groups throughout at least 7 days after the muscle injured, since the pain and blood CK levels might peak between 48 and 72 h after the EIMD protocol. Furthermore, the experiments might also need to account for the volume and intensity of the muscle damage protocol and for this, the trial must be extremely well applied, ideally equalizing total work performed during the muscle damage inducing exercise protocol.

The effects of chronic caffeine supplementation and its effects on EIMD and recovery may also be an important area of focus for future. A study with rats demonstrated that chronic caffeine supplementation (1 mg·mL^−1^ diluted in tap water) for 30 days resulted in lower blood CK activity, fewer damaged muscle cells, and a lower amount of inflammatory cells in both sedentary and trained animals after the last exercise session [84]. Furthermore, 4 weeks of coffee consumption in mice prior to injury induced by cardiotoxin has been shown to increase muscle regenerative capacity, which has been attributed to cell proliferation marker Ki67 and an increased quantity of embryonic myosin heavy chain, a marker of immature myotubes [85]. Whilst the results of previous work are promising, they should be interpreted with caution given that these findings have only been demonstrated in animal models. In addition, coffee contains several other substances in addition to caffeine, such as chlorogenic acids, which may contribute the effects independent of caffeine. Moreover, muscle damage was induced by cardiotoxin injection, which differs in magnitude of muscle damage when compared to EIMD. Whether caffeine is able to acutely stimulate satellite cells proliferation and assist in the regeneration process after EIMD is still a matter of speculation and should be further investigated.

This review also highlights some methodological issues that should be considered in future studies, such as better methodological control of the study quality by PEDro scale, standardizing the caffeine treatment dose and, when possible, optimizing and opting for the cross-over experimental design with a longer wash-out period, allowing a complete reestablishment of the total body homeostasis between test conditions.

## 5. Conclusions

The present systematic review found 14 studies that evaluated the effect of caffeine on indirect muscle damage markers, including blood markers, pain perception, and strength performance. Of the nine studies using blood markers, six indicated that caffeine administered pre (five studies) or post (one study) an EIMD protocol does not cause more muscle damage, considering CK circulating levels, but this should be interpreted with caution due to the limitations of using CK as a marker of muscle damage, as discussed herein. Caffeine ingestion may result in lower pain perception (as shown in four of six studies included in this systematic review). The effects of caffeine on muscle strength following EIMD is still inconclusive, due to the limited number of studies and conflicting results. The limited and controversial evidence prevents accurate conclusions regarding the effects of acute caffeine consumption on the attenuation of muscle damage when used prior to an EIMD protocol. Caffeine consumed following EIMD however may acutely relieve some symptoms, attenuating pain perception and potentially increasing strength. Based on the current review and on its well described ergogenic effects, caffeine supplementation may be a valid strategy for athletes who need to recover between strenuous training sessions or competitions.

## Figures and Tables

**Table 1:** Characteristics of the studies and main results.

Study	Training Status	Sample	Age	Caffeine Consumption	Study Design	Muscle Damage Protocol	Supplementation Protocol	Findings
								(Caf × Pla)
Caldwell et al. [25]	Recreational cyclists	*n* = 30;	46 ± 11	~230 mg/day	2 parallel groups	164 km of cycling	2 daily doses for 4 days after (8 doses of 3 mg/kg)	↓DOMS in Caffeine group 24 h after EIMD.
		(25M; 5F)			(Caf; Pla)			
Cameron et al. [32]	Soccer athletes	*n* = 22M	26–31	Non described	3 parallel groups	VDR + Yo-Yo IRT	pre-exercise	↑∆CK in Caffeine group; No difference between groups for CKMB and LDH.
					(Caf; Pla; Con)		(1 dose of 5 mg/kg)	
Chen et al. [27]	College athletes	*n* = 20;	M = 21.1 ± 1.1; F = 20.4 ± 1.2	<200 mg/wk	Crossover	Downhill running	24 h or 48 h post-exercise	↑recovery of MVIC and ↓ DOMS in Caffeine group 48 h after EIMD.
		(10M; 10F)			(After 24 h)	(30-min)	(1 dose of 6 mg/kg)	
Ferreira et al. [28]	Physically active	*n* = 20M	25.2–26.2	±72 mg/day	2 parallel groups	13 × 30 s sprint standard bicycle	Pre-exercise	↑CK 24 and 48 h after EIMD in Placebo group.
					(Caf; Pla)		(1 dose of 5 mg/kg)	
Green et al. [33]	Physically active	*n* = 16	24.3 ± 4.3	Usual consumers	Crossover	Eccentric contraction	Pre, 24 h post-exercise	↑MVDC in Caffeine group; No difference between groups for DOMS and MVIC.
		(8M; 8F)			(After 1 week)	(Quadriceps)	(2 doses of 6 mg/kg)	
Hurley et al. [34]	Resistance-trained	*n* = 12M	20 ± 1	Low consumers	Crossover	Eccentric contraction	Pre, 24–120 h post-exercise	↓DOMS on the 2–3 day in Caffeine group; No difference between groups for CK.
					(After 1 week)	(Elbow flexors)	(6 doses of 5 mg/kg)	
Kazman et al. [35]	Non described	*n* = 35	27,2 ± 8	Non described	Crossover	60-min walking and	Pre-exercise	No difference between groups for CK.
		(29M; 6F)			(After 1 week)	5-min step/squat	(1 dose of 7.5 mg/kg)	
Machado et al. [36]	Soccer athletes	*n* = 15M	18.4 ± 0.8	<100 mg/day	Crossover	Full body strength session	Pre-exercise	No difference between groups for CK and LDH.
					(After 1 week)		(1 dose of 4.5 mg/kg)	
Mahdavi et al. [37]	Basketball athletes	*n* = 26F	24.22 ± 2.65	116.88 mg/day	Crossover	Wingate test	Pre-exercise	No difference between groups for CK, MDA, and TAC
					(After 1 week)	(30-sec)	(1 dose of 5 mg/kg)	
Maridakis et al. [30]	No experience Strength training	*n* = 10F	21.3 ± 1.6	55.1 ± 30.9 mg/day	Cross-over	Eccentric contraction (Quadriceps)	Pre, 24 h or 48 h post-exercise	↓DOMS in Caffeine group; No difference between groups for MVIC.
					(After 24 h or 48 h)		(2 doses of 5 mg/kg)	
Ribeiro et al. [38]	Handball athletes	*n* = 6M	21.6 ± 2.9	~60 mg/day	Cross-over	Vertical jumps	Pre-exercise	↑vertical jump in Caffeine group; no difference between groups for CK and LDH.
					(After 1 week)	(4 sets of 30-sec)	(1 dose of 6 mg/kg)	
Stadheim et al. [39]	Elite cross-country skiers	*n* = 8M	20,0 ± 1,0	<150 mg/day	Cross-over	Double poling ergometer (10-min)	1 dose pre-exercise	↑test performance, ↑CK, ↑DOMS in Caffeine groups (3 mg/kg–4.5 mg/kg)
					(After 6 days)		(3 mg/kg or 4.5 mg/kg)	
Park et al. [29]	Non described	*n* = 13	25.5 ± 3.3	213 ± 151 mg/day	Cross-over	Eccentric contraction (Quadriceps)	24–48 h post-exercise	No difference between groups for MVIC.
		(4M; 9F)			(After 2 week)		(2 doses of 6 mg/kg)	
Vimercatt et al. [40]	Physically active	*n* = 15M	19 ± 1	Non described	Cross-over	Treadmill running	1 dose pre-exercise	No difference between groups for CK, LDH, ALT, and AST.
					(After 2 week)	(60-min)	(4.4 mg/kg or 5.5 mg/kg)	

ALT = alanine aminotransferase; AST = aspartate aminotransferase; Caf = caffeine Group; CK = creatine kinase; CKMB = creatine kinase MB isoform; DOMS = delayed onset muscle soreness; EIMD = exercise-induced muscle damage; F = female; LDH = lactate dehydrogenase; M = male; MDA = malondialdehyde; MVDC = maximum voluntary dynamic contraction; MVIC = maximum voluntary isometric contraction; *n* = sample size; Pla = placebo group; TAC = total antioxidant capacity; VDR = variable distance run protocol; YoYo IRT = Yo-Yo intermittent recovery test; ↓ = statistically significant decrease; ↑ = statistically significant increase.

**Table 2:** Assessment of the methodological quality of studies using the PEDro scale.

Study						Criteria						PEDro Score
	1*	2	3	4	5	6	7	8	9	10	11	
Caldwell et al. [25]	1	1	1	1	1	1	1	1	1	1	1	10
Cameron et al. [32]	0	1	1	1	1	1	1	0	0	1	1	8
Chen et al. [27]	1	1	1	1	1	1	1	1	1	1	1	10
Ferreira et al. [28]	0	1	1	1	1	1	1	1	1	1	1	10
Green et al. [33]	1	1	1	1	1	1	1	1	1	1	1	10
Hurley et al. [34]	0	1	1	1	1	1	1	1	1	1	1	10
Kazman et al. [35]	1	1	1	1	1	1	1	1	1	1	1	10
Machado et al. [36]	0	1	1	1	1	1	1	1	1	1	1	10
Mahdavi et al. [37]	0	1	1	1	1	1	1	1	1	1	1	10
Maridakis et al. [30]	1	1	1	0	1	1	1	1	1	1	1	9
Ribeiro et al. [38]	0	1	1	1	1	1	1	1	1	1	1	10
Stadheim et al. [39]	0	1	1	1	1	1	1	1	1	1	1	10
Park et al. [29]	1	1	1	1	1	1	1	1	1	1	1	10
Vimercatt et al. [40]	0	1	1	1	1	1	1	1	1	1	1	10

Criteria 1* = the eligibility criterion is not scored for being related to external validity and, therefore, does not reflect the quality dimensions assessed by the PEDro Scale.

## Data Availability

The data presented in this study are available upon request from the corresponding author.
